# Case report: Bridging limbic network epilepsy with psychiatric, memory, and sleep comorbidities: case illustrations of reversible psychosis symptoms during continuous, high-frequency ANT-DBS

**DOI:** 10.3389/fnetp.2024.1426743

**Published:** 2024-08-08

**Authors:** Lydia Wheeler, Samuel E. Worrell, Irena Balzekas, Jordan Bilderbeek, Dora Hermes, Paul Croarkin, Steven Messina, Jamie Van Gompel, Kai J. Miller, Vaclav Kremen, Gregory A. Worrell

**Affiliations:** ^1^ Bioelectronic Neurophysiology and Engineering Laboratory, Department of Neurology, Mayo Clinic, Rochester, MN, United States; ^2^ Department of Physiology and Biomedical Engineering, Mayo Clinic, Rochester, MN, United States; ^3^ Department of Neurologic Surgery, Mayo Clinic, Rochester, MN, United States; ^4^ Departments of Psychiatry and Psychology, Mayo Clinic, Rochester, MN, United States; ^5^ Department of Radiology, Mayo Clinic, Rochester, MN, United States; ^6^ Czech Institute of Informatics, Robotics, and Cybernetics, Czech Technical University, Prague, Czechia

**Keywords:** limbic network, Epilepsy, seizure, psychosis, ANT-DBS

## Abstract

The network nature of focal epilepsy is exemplified by mesial temporal lobe epilepsy (mTLE), characterized by focal seizures originating from the mesial temporal neocortex, amygdala, and hippocampus. The mTLE network hypothesis is evident in seizure semiology and interictal comorbidities, both reflecting limbic network dysfunction. The network generating seizures also supports essential physiological functions, including memory, emotion, mood, and sleep. Pathology in the mTLE network often manifests as interictal behavioral disturbances and seizures. The limbic circuit is a vital network, and here we review one of the most common focal epilepsies and its comorbidities. We describe two people with drug resistant mTLE implanted with an investigational device enabling continuous hippocampal local field potential sensing and anterior nucleus of thalamus deep brain stimulation (ANT-DBS) who experienced reversible psychosis during continuous high-frequency stimulation. The mechanism(s) of psychosis remain poorly understood and here we speculate that the anti-epileptic effect of high frequency ANT-DBS may provide insights into the physiology of primary disorders associated with psychosis.

## 1 Introduction

Epilepsy is a common neurologic disease characterized by recurrent seizures that impact over 60 million people worldwide (Institute of Medicine Committee on the Public Health Dimensions of the Epilepsies et al., 2012; Devinsky et al., 2018). In addition to seizures, the quality of life for people with epilepsy (PWE) can be markedly affected by psychiatric and neurologic comorbidities (Institute of Medicine Committee on the Public Health Dimensions of the Epilepsies et al., 2012). Epilepsy is a network circuit disorder with dysregulation of specific brain networks underlying sporadic seizures and chronic comorbidities (Institute of Medicine Committee on the Public Health Dimensions of the Epilepsies et al., 2012; Spencer, Gerrard, and Zaveri, 2018; Lehnertz, Bröhl, and Wrede, 2023). Anti-seizure medications are the mainstay of epilepsy therapy, but over 1/3 PWE have drug-resistant epilepsy (Kwan et al., 2011) and continue to have seizures despite taking daily medications.

Mesial temporal lobe epilepsy (mTLE) is one of the most common epilepsies. It is defined by focal seizures originating from mesial temporal structures (J. Engel Jr, 2001; Berg, 2008) that comprise part of the limbic circuitry (Papez, 1937; MacLean, 1949; Child and Eduardo, 2013) with seizures involving the amygdala, hippocampus, and parahippocampal neocortex. mTLE (Berg, 2008) is a common limbic circuit disorder with disabling seizures and a high incidence of interictal psychiatric (Nadkarni, Arnedo, and Devinsky, 2007; Kanner, 2009; Kanner, Ribot, and Mazarati, 2018), memory (B. Bell et al., 2011; McAuley et al., 2010), and sleep (MMS) disturbances (Devinsky et al., 2018; Moore et al., 2021; Garg, Charlesworth, and Shukla, 2022). Destructive surgical procedures are proven treatments for drug-resistant mTLE (Wiebe et al., 2001; Engel Jr et al., 2012). Unfortunately, many people with drug-resistant mTLE are poor surgical candidates because the destruction of the circuit responsible for their seizures would negatively impact memory (Engel et al., 2012; M. L; Bell et al., 2009). The same limbic circuitry responsible for mTLE continues to perform vital functions when it is not producing sporadic, disabling seizures. These treatment challenges are more pronounced for people with normal structural imaging (M. L. Bell et al., 2009), bilateral mTLE (Squire, 2009), or high baseline memory performance. This has stimulated interest in non-destructive, reversible options like electrical brain stimulation (Fisher and Ana, 2014).

**FIGURE 1:**
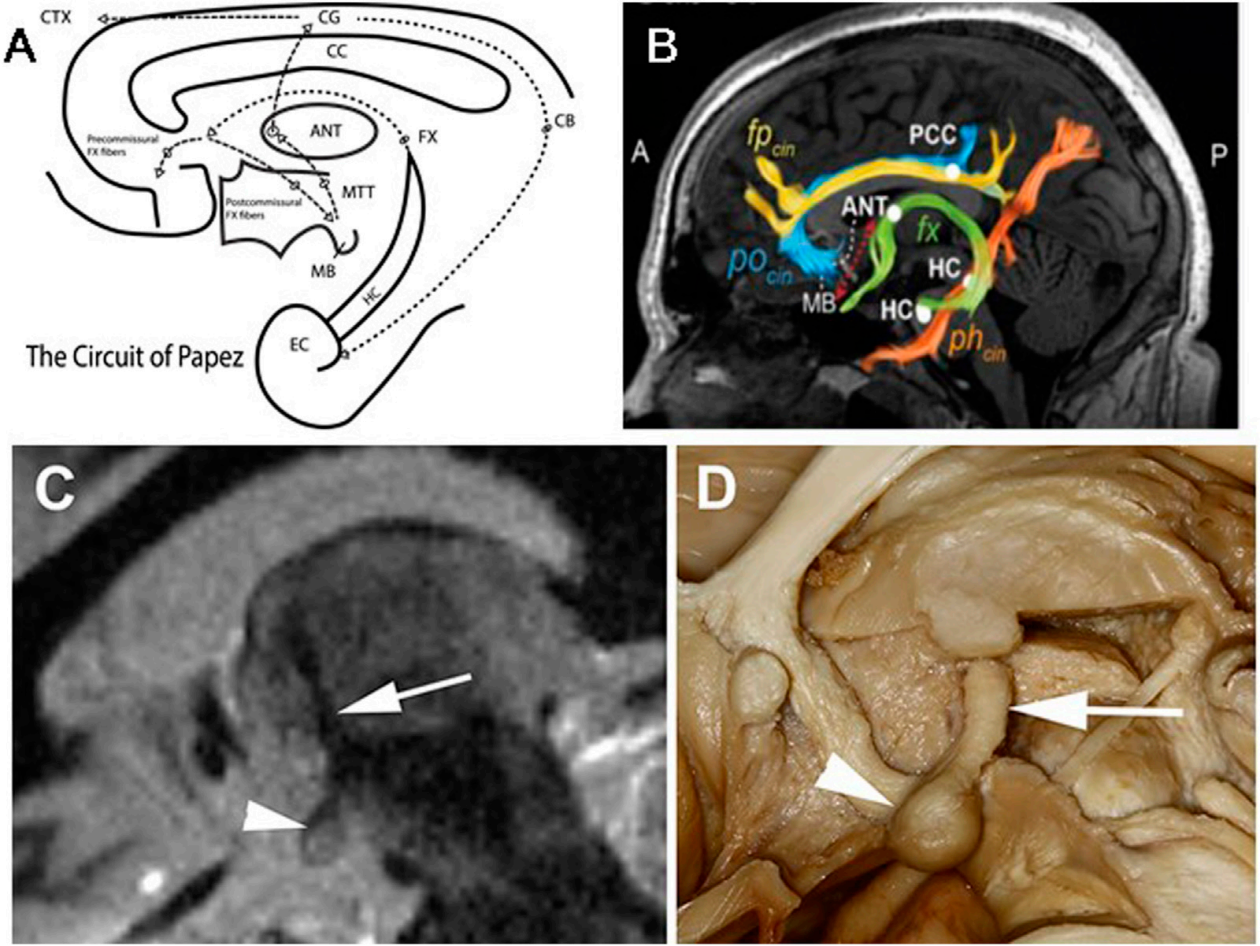
Limbic Circuit of Papez and MacLean: McLean modified Papez version of the limbic circuitry and emphasized the amygdala and septum in addition to hippocampus [20]. **(A)** Schematic of Limbic Circuit highlighting the closed-loop pathway from the entorhinal cortical (EC) input to hippocampus (HC). The outflow of HC via the Fornix (fx) fibers spans the corpus callosum (CC) and divides into pre- (terminate in Septal Nuclei (SN)) and post- (terminate in the mammillary bodies (MB)) CC commissural fibers. The post-commissural fibers project through the hypothalamus to the MB, and MB fibers project via mammillary thalamic tract (MTT) to the anterior nucleus of the thalamus (ANT). (Note: The MTT is widely used for direct targeting of the ANT for ANT-DBS). The ANT output via anterior thalamocortical fibers to the anterior Cingulate Gyrus and back to EC and HC via the Cingulum. **(B)** Diffusion tensor imaging (DTI) demonstrating white matter tracks discussed above. **(C)** Fast gray matter acquisition T1 inversion recovery (FGATIR) MRI sequence visualizing the MB, MTT and ANT. **(D)** Human brain dissection demonstrating the MB and MTT connecting MB and ANT. **(B)** modified from Ojeda Valencia, Gabriela, et al. “Signatures of Electrical Stimulation Driven Network Interactions in the Human Limbic System.” Journal of Neuroscience: 43, no. 39 (27 September 2023): 6697–6711. **(C,D)** modified from Grewal, S., et al. “Fast Gray Matter Acquisition T1 Inversion Recovery MRI to Delineate the Mammillothalamic Tract for Preoperative Direct Targeting of the Anterior Nucleus of the Thalamus for Deep Brain Stimulation in Epilepsy.” Neurosurgical Focus 45, no. 2 (2018).

The human limbic circuit is a complex system involving multiple brain structures and is essential for regulating emotional processing, memory processing, and sleep and alertness. The circuit is generally considered to include the amygdala, hippocampus, anterior nucleus of the thalamus, hypothalamus, cingulate cortex and fornix (Figure 1). The amygdala (AMG) is critical for processing emotions and how memories are influenced by emotion (Paré and Headley, 2023). The HPC is critical for encoding memories in thalamocortical networks including long-term, autobiographical memory (Baker and Zeman, 2017; Lemesle et al., 2021). The anterior nucleus of the thalamus (ANT) is crucial for regulating memory, sleep, and alertness (Szabó et al., 2022). The hypothalamus regulates homeostatic functions. The cingulate cortex plays a role in emotional and cognitive processing and the fornix (FRX) is a significant output tract from the hippocampus.

### 1.1 Ictal and interictal epileptiform discharges mTLE

The symptoms and signs of TLE seizures have appeared in medical literature throughout history (Temkin, 1994). Hughlings Jackson, in the 19th century, described intellectual or “dreamy state” temporal lobe seizures (Hughlings-Jackson, 1888; Hogan and Kitti, 2003). The seizures in people with mTLE are primarily diurnal, occurring during the daytime (Durazzo et al., 2008; Mivalt et al., 2023).

Interictal epileptiform spikes (IES) and pathological high-frequency oscillations (Worrell and Gotman, 2011) are interictal biomarkers of the epileptogenic brain with ultradian, circadian, and infradian periodicities (Baud et al., 2018; Karoly et al., 2021). Interestingly, the IES are increased in slow-wave sleep but seizures are more likely in wakefulness in mTLE (Kremen et al., 2024).

### 1.2 Interictal comorbidities of mTLE

It is widely appreciated that mTLE is associated with Memory, Mood, and Sleep (MMS) Epilepsy Comorbidities. There are well-known but poorly understood complex bidirectional interactions between epilepsy, sleep (Grigg-Damberger and Foldvary-Schaefer, 2021; Moore et al., 2021), mood (Bølling-Ladegaard et al., 2023), and memory (Blake et al., 2000).

Verbal and spatial memory deficits are a common problem in mTLE, contributing to one of the major comorbidities affecting quality of life (Milner, 1968; L. R; Squire and Zola-Morgan, 1991; McAuley et al., 2010). Multiple studies show that IES in TLE can impair memory task performance (Kleen et al., 2013; Matsumoto et al., 2013; Horak et al., 2017). Accelerated long-term forgetting describes difficulties that people with epilepsy have in retaining new information over extended periods of time (Blake et al., 2000; Baker and Zeman, 2017; Helmstaedter et al., 2019). Seizures and interictal epileptiform spikes/sharp waves (IES) may interfere with the neural process by which memories are stabilized and stored for long-term use, i.e., the memory consolidation process (Blake et al., 2000). The possibility that mTLE “hijacks” the physiological process of memory and consolidation of engrams underlying the natural progression of mTLE is interesting to consider and suggests that post-ictal disruption of consolidation may be a viable treatment strategy (Beenhakker and Huguenard, 2009; Bower et al., 2015; 2017).

Sleep disruption is common in epilepsy (Moore et al., 2021; Garg, Charlesworth, and Shukla, 2022) and can be exacerbated by thalamic deep brain stimulation (Voges et al., 2015; Szabó et al., 2022; F; Mivalt et al., 2022; Kremen et al., 2024).

People with epilepsy have high rates of psychiatric comorbidity and there is a well-established bidirectional relationship (Kanner, Ribot, and Mazarati, 2018; Bølling-Ladegaard et al., 2023). The lifetime prevalence of major depressive disorder and anxiety-related disorders in epilepsy is 6% and 22%, higher than in the general population (Tellez-Zenteno et al., 2007). The severity of depression and anxiety symptoms can be better predictors of quality of life in epilepsy than seizure frequency (Boylan et al., 2004; Johnson et al., 2004).

Psychosis is a less common but clinically significant phenomenon in mTLE (Nadkarni, Arnedo, and Devinsky, 2007). Psychosis symptoms in mTLE resemble those seen in primary psychiatric disorders such as schizophrenia, including hallucinations (typically auditory but also visual), delusions, and paranoid thoughts. Psychosis is associated with seizures is referred to as “ictal or post-ictal psychosis,” occurring during or closely following a seizure. Interictal psychosis occurs independently of seizure activity (Leutmezer et al., 2003; Hilger et al., 2013).

### 1.3 Neuromodulatory treatment for drug-resistant mTLE

Early investigations using thalamus deep brain stimulation began with anterior nucleus of thalamus deep brain stimulation (ANT-DBS) (Cooper and Upton, 1985) and centromedian nucleus of thalamus (CMT-DBS) (Velasco et al., 1987). Later controlled trials targeting vagus nerve stimulation (VNS) (E. Ben-Menachem et al., 1999; Ben-Menachem, 2002; R. S; Fisher, 2012), deep brain stimulation targeting the anterior nucleus of the thalamus (ANT-DBS) (R. Fisher et al., 2010; Salanova et al., 2021), and responsive neural stimulation (RNS) (Morrell and RNS System in Epilepsy Study Group, 2011) lead to class-I evidence for drug-resistant mTLE. But patients rarely achieve long-term, >1 year, seizure freedom. The pivotal SANTE (Stimulation of ANT for Epilepsy) trial proved duty cycle, high-frequency stimulation (145 Hz; 1 min on and 5 min off) reduced seizures. However, ANT-DBS with SANTE stimulation parameters rarely yields seizure-free outcomes and can exacerbate mood, memory, and sleep (MMS) comorbidities (Voges et al., 2015; Tröster et al., 2017; Szabó et al., 2022). Despite increased mood and memory complaints in SANTE standard neuropsychological assessments did not detect a change (Tröster et al., 2017).

**FIGURE 2:**
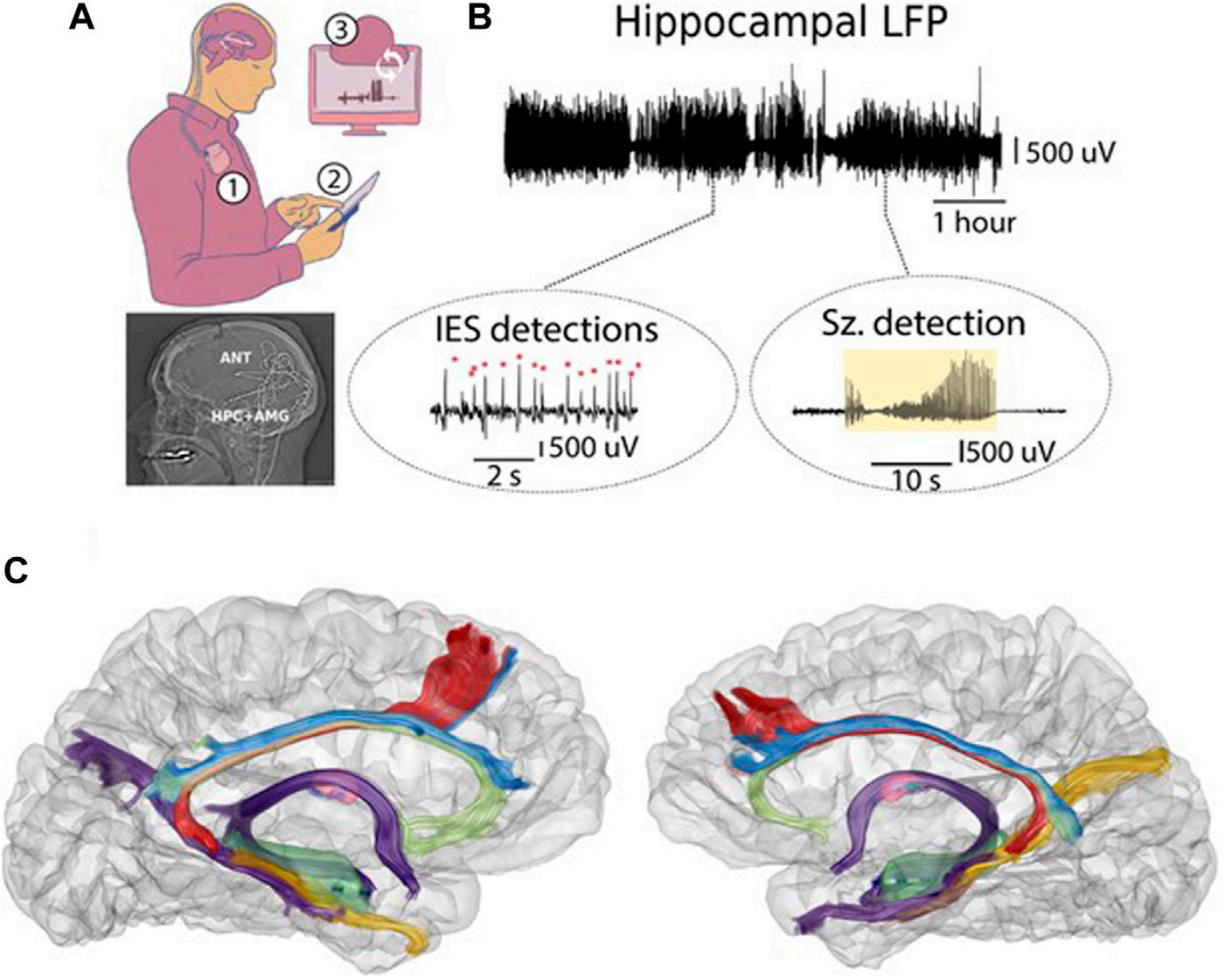
Human limbic circuit electrophysiology. Continuous LFP streaming was used to track mesial temporal lobe and ANT electrophysiology in people with mTLE living in their natural home environment. **(A)** Synchronized bidirectional communications between patients, implantable neural sensing-stimulation devices, mobile devices, and cloud computing infrastructure enable monitoring electrophysiology. Top) Implanted neural sensing and stimulation device in subclavicular pocket (1). The implanted devices have bidirectional communication with the mobile device (2) and cloud environment (3). Bottom) Lateral x-ray post-implant showing the 4 leads implanted in bilateral ANT and AMG-HPC. **(B)** Top) Continuous LFP over multiple hours. Bottom) Circles highlight automated IES and seizure detections on mobile devices (tablet computers). **(C)** Co-registration of CT and MRI-DTI demonstrating the posterior approach to the AMG-HPC and ANT for brain sensing and stimulation electrodes.

Relatively little is known about ANT-DBS parameter optimization (R. S. Fisher, 2023; Lundstrom and Gregg, 2023; McIntyre et al., 2006) and assessment of MMS comorbidities have largely relied on sparse data collected at clinic visits. Seizure counts have relied on patient diaries that are known to be inaccurate (Elger, 2014; Morrell, 2014; Osorio, 2014; Elger and Hoppe, 2018). Advances in MRI imaging have significantly improved the targeting for ANT-DBS (Figure 1) (Grewal et al., 2018; Järvenpää et al., 2020). In summary, optimal ANT-DBS parameters for mTLE remain unknown. Emerging device technologies are overcoming the gaps and providing dense behavioral tracking and accurate seizure diaries (Figure 2) (V. Kremen et al., 2018; Sladky et al., 2022; F; Mivalt et al., 2022; Kremen et al., 2018; Filip Mivalt, Sladky, et al., 2023; Gregg et al., 2020) to help optimize ANT-DBS parameters.

## 2 Case report of two patients undergoing continuous high-frequency ANT-DBS

Here, we describe two subjects implanted with an investigational device (Medtronic Plc. Summit RC + S™) with continuous hippocampal local field potential (LFP) sensing, enabling accurate seizure diaries (Sladky et al., 2022; V; Kremen et al., 2018). The patients each had over 1 year of weekly contact with the research and clinical teams to assess device connectivity, signal fidelity, seizure frequency, and clinical status. This close monitoring yielded the following case narratives which are high-level summaries of both in-person and remote clinical assessments and patient self-reported symptoms. Although neuropsychological testing was performed at fixed, pre-determined intervals during the study, additional quantitative assessments were not performed at the time of the patients’ psychotic symptoms. Both patients developed reversible psychosis with continuous high-frequency ANT-DBS. The psychosis resolved with the transition to duty cycle stimulation (1 min on and 5 min off) as used in the SANTE trial.

### 2.1 Patient 1

A 57-year-old, ambidextrous female presented with a 40-year history of epilepsy, depression, and anxiety. At 9 years old, she had a significant head trauma associated with a brief loss of consciousness. No imaging was performed at the time, and she made a complete recovery. She did well in school, but at age 14 years she began having sporadic episodes of déjà vu lasting less than a minute and without loss of awareness. The spells became more intense in college, with intense fear, depersonalization, and false memories lasting 1–2 min in duration. By age 21, the spells commonly progressed to generalized tonic-clonic convulsions. She was started on anti-seizure medications that eliminated the convulsive seizures, but she continued to have seizures with loss of awareness despite multiple anti-seizure medications (ASMs) trials.

She reported a long-standing history of anxiety, stating she awakened each day with fear about having a seizure. She had a history of depression accompanied by low mood, anhedonia, decreased appetite, tearfulness, and insomnia. She had no prior history of mania, hypomania, or psychosis.

She underwent a comprehensive evaluation for drug-resistant epilepsy.

1) Laboratory studies were unremarkable, including an autoimmune epilepsy panel.
2) MRI was unremarkable except for subtle indistinct bilateral hippocampal internal architecture and possible left amygdala enlargement.
3) EMU: Video-EEG monitoring showed frequent bilateral temporal intermittent rhythmic delta activity and independent, left greater than right, temporal epileptiform sharp waves. Focal impaired aware seizures (FIAS), originating from both the left and right temporal head regions, were recorded.
4) Stereo EEG: Four left hippocampal onset FIAS and two right hippocampal onset FIAS were recorded.
5) Presentation at epilepsy surgery conference: Consensus recommendation for neuromodulation with bilateral mesial temporal RNS, ANT-DBS, or investigational study.
6) The patient was consented for the FDA-IDE (https://clinicaltrials.gov/study/NCT03946618) and underwent placement of Medtronic RC + S™ device with 4 leads (bilateral anterior nucleus of thalamus and bilateral amygdala-hippocampus).

As part of the FDA-IDE protocol, she was admitted to hospital epilepsy monitoring unit (EMU) for neurostimulator programming prior to initiation of long-term ambulatory stimulation. She tolerated multiple trial stimulation paradigms and did report a decrease in seizures with ANT 2 Hz stimulation, but continued COVID-19-related anxiety affected her appetite and mood, and continued irritation and stable dysphoria. A subsequent trial of high-frequency ANT-DBS (*continuous* 145 Hz, 90 PW, 3–5 mA) significantly exacerbated her COVID-19-related anxiety, anorexia, and dysphoria. Within a month of initiating continuous high-frequency ANT-DBS, she reported increasing anxiety and worsening insomnia and mood. She continued to lose weight. A trial of mirtazapine for her anxiety, insomnia, and decreased appetite was not helpful. She subsequently reported “daymares” associated with dissociative symptoms and a sense of inability to distinguish dreams from reality. Transition to duty cycle HF ANT-DBS (SANTE parameters) quickly improved her symptoms of insomnia, anxiety, daymares, dissociation, and difficulty distinguishing between dreaming and reality.

Subsequent trials of low frequency (2 Hz continuous 3 mA, 200 μs PW) were associated with decreased frequency and severity of seizures as well as subjective and clinically assessed improved mood and anxiety. She reports improvement in her sleep, although she believes her dreams have been disrupted. The patient continues to maintain stable mood, weight, and sleep (Kremen et al., 2024).

### 2.2 Patient 2

A 41-year-old, right-handed female with a long-standing history of epilepsy, major depression, and anxiety. She had no risk factors for epilepsy. At the age of 37 years old, she had an unprovoked generalized tonic clonic (GTC) from sleep. In the subsequent months, she developed FIAS with staring, behavioral arrest, and unresponsiveness occurring as often as weekly. With ASMs, she no longer had GTC but continued to have weekly FIAS. She also received a trial of immunotherapy after elevated GAD65 was identified in CSF and serum without benefit. She had a VNS implanted that decreased seizures by approximately 25%, but despite ASM and VNS optimization, she continued to have multiple FIAS each month.

Her mood symptoms began about 5–6 years before her initial seizure. Her anxiety revolved around concerns about her children, the future, her health, and seizures. Her depression was often accompanied by insomnia, decreased appetite, tearfulness, and hopelessness. She had no prior symptoms consistent with hypomania, mania, or psychosis although she had a history of a 5-day episode of hyperactive delirium during her sEEG monitoring which resolved with discontinuation of anticholinergic medications. Her neuropsychology testing showed deficits in delayed memory for both verbal and visual information, referable to bilateral mesial temporal dysfunction.

1) Laboratory studies were remarkable for elevated GAD-65 in serum and CSF.
2) MRI showed an increased T2 signal in the left hippocampus but with normal volumetric measurements.
3) EMU: Video-EEG monitoring showed bilateral independent, left greater than right, temporal epileptiform sharp waves. FIAS seizures originating from the left and right temporal head regions were recorded.
4) Stereo EEG: A single left hippocampal onset seizure was recorded. The evaluation was complicated by a period of agitation and psychosis requiring Haldol.
5) Presentation at epilepsy surgery conference: Consensus recommendation for neuromodulation with RNS or possibly ANT-DBS, but given the findings during sEEG, ANT stimulation was considered a higher risk of side effects. She was a possible candidate for left mesial temporal destructive procedures with laser interstitial thermal therapy (LITT) (Youngerman et al., 2023) or left anterior temporal lobectomy with amygdala-hippocampectomy (Wiebe et al., 2001). However, given the normal hippocampal volume and left-brain dominance the likely impact on verbal memory was not acceptable to the patient.
6) The patient was consented for FDA-IDE (https://clinicaltrials.gov/study/NCT03946618) and underwent placement of Medtronic RC + S™ device with 4 leads (bilateral anterior nucleus of thalamus and bilateral amygdala-hippocampus).

One month later as part of the FDA-IDE protocol, she was admitted for neurostimulator programming prior to initiation of long-term therapeutic ANT-DBS. With continuous 145 Hz stimulation, 4 mA, 90 µs cycling, the patient had an approximately 1-h episode with elevated respiratory rate, dyspnea, disorientation, and visual hallucinations. She became anxious and described a visual hallucination of being in a long room with many beds. She was considerably agitated and concerned about her family. She repeatedly called her husband, who helped calm her, but she continued to perseverate with concerns about her children. There was no change in the scalp EEG or the ANT and HPC recordings during the event, and this was different from her typical seizure semiology. Her stimulation was adjusted to continuous 2 Hz with the same current, and she rapidly improved. Later in the trial she also received duty-cycle high frequency ANT-DBS (SANTE parameters) without side effects.

With ANT-DBS, her seizure frequency decreased 90% from baseline and 50% from VNS, and she reported decreased seizure severity with both low and high frequency duty cycle ANT-DBS. She continues to report substantially improved functional status, and no worsening of depressive or anxiety symptoms throughout continued responsive ANT-DBS stimulation. No other episodes of psychosis reported. Each of her mood, memory, and sleep symptoms improved with extended time without seizures.

## 3 Discussion

High-frequency duty cycle ANT-DBS is a proven therapy for mTLE (R. Fisher et al., 2010). The precise neurobiological mechanisms for the effectiveness in reducing seizures remain poorly understood. Similarly, the mechanism(s) that underlie psychosis in TLE are not well understood (Nadkarni, Arnedo, and Devinsky, 2007). Here we were able to rule out the patients’ habitual mTLE seizures with continuous hippocampal LFP streaming (Figure 2).

New-onset psychiatric issues are not uncommon following ANT-DBS, with depression being the most common (R. Fisher et al., 2010; Salanova et al., 2015; Tröster et al., 2017). Interestingly, an early study even reported improvement in seizures and psychosis with ANT-DBS (Upton et al., 1985). In a study by Järvenpää et al. (Järvenpää et al., 2018), 2 of 22 patients (9%) developed psychosis following ANT-DBS that resolved with programming changes. Notably, there were no group-level declines in mood or memory status following ANT-DBS in the open-label follow-up of the SANTE cohort (Tröster et al., 2017).

The precise neurobiological mechanisms that underlie psychosis in TLE are not fully understood. Hypotheses include neurotransmitter imbalance, especially serotonin and dopamine, inflammatory cytokines, and genetic predispositions. High-frequency ANT-DBS desynchronizes HPC LFP activity (Stypulkowski et al., 2014; Yu et al., 2018) and fragments sleep (Voges et al., 2015; Szabó et al., 2022; Kremen et al., 2024) that could contribute to psychosis. Disorders of neural synchrony have been proposed as a mechanistic model for schizophrenia (Uhlhaas and Singer 2010). Additionally, the effect of ANT-DBS on sleep may be related to its impact on surrounding thalamic nuclei. In particular, HF stimulation of central laminar thalamus has been shown to promote arousal and would likely disrupt sleep (Redinbaugh et al., 2020). In the future, it may be possible to further dissect the underlying mechanism of ANT-DBS related psychosis.

In the current study, limiting high-frequency ANT-DBS to duty cycle paradigms (1 min on and 5 min off) appeared to be better tolerated. In the setting of electrical brain stimulation, it is essential to recognize psychiatric side effects early, adjust stimulation parameters, and consider pharmacologic interventions such as antipsychotic medications (de Toffol et al., 2018). It is often difficult to rule out the role of seizure activity in the development of psychosis in people with TLE. However, in this report with continuous LFP tracking and seizure detection we were able to demonstrate that seizures did not explain their psychosis.

In conclusion, ANT-DBS in people with mTLE is not uncommonly associated with exacerbation, or even new onset of psychiatric symptoms (Tröster et al., 2017). It is critical to quickly recognize the development of new symptoms or exacerbation of prior symptoms. Fortunately, here the symptoms were responsive to programming changes in these two patients.

## Data Availability

The original contributions presented in the study are included in the article/supplementary material, further inquiries can be directed to the corresponding authors.
